# Retrospective 25-year follow-up of treatment outcomes in angle Class III patients: Early versus late treatment

**DOI:** 10.1007/s00056-016-0076-7

**Published:** 2017-02-20

**Authors:** B. Wendl, A. P. Muchitsch, H. Winsauer, A. Walter, H. Droschl, N. Jakse, M. Wendl, T. Wendl

**Affiliations:** 1 Clinical Department of Oral Surgery and Orthodontics, Medical University Graz, Billrothgasse 4, 8036 Graz, Austria; 2 Private Practice, Bregenz, Austria; 3 Department of Orthodontics and Dentofacial Orthopedics, International University of Catalonia, Barcelona, Spain; 4 Institute of Software Development and Biomedical Engineering, Technical University Graz, Graz, Austria

**Keywords:** Class III treatment, Early treatment, Late treatment, Chincup, Klasse-III-Therapie, Frühtherapie, Spättherapie, Kopf-Kinn-Kappe

## Abstract

**Objectives:**

To assess early versus late treatment of Class III syndrome for skeletal and dental differences.

**Methods:**

Thirty-eight Class III patients treated with a chincup were retrospectively analyzed. Baseline data were obtained by reviewing pretreatment (T0) anamnestic records, cephalograms, and casts. The cases were assigned to an early or a late treatment group based on age at T0 (up to 9 years or older than 9 years but before the pubertal growth spurt). Both groups were further compared based on posttreatment data (T1) and long-term follow-up data collected approximately 25 years after treatment (T2).

**Results:**

Early treatment was successful in 74% and late treatment in 67% of cases. More failures were noted among male patients. The late treatment group was characterized post therapeutically by significantly more pronounced skeletal parameters of jaw size relative to normal Class I values; in addition, a greater skeletal discrepancy between maxilla and mandible, higher values for mandibular length, Cond-Pog, ramus height, overjet, anterior posterior dysplasia indicator (APDI), lower anterior face height, and gonial angle were measured at T1. The angle between the AB line and mandibular plane was found to be larger at T0, T1, and T2, as well as more pronounced camouflage positions of the lower anterior teeth at T0. The early treatment group was found to exhibit greater amounts of negative overjet at T0 but more effective correction at T1.

**Conclusions:**

Early treatment of Class III syndrome resulted in greater skeletal changes with less dental compensation.

## Zusammenfassung

**Zielsetzung:**

In dieser Arbeit sollten mögliche Unterschiede zwischen Früh- und Spättherapie beim Klasse-III-Syndrom ermittelt werden.

**Material und Methode:**

Es erfolgte eine retrospektive Analyse von 38 therapierten Patienten mit Angle-Klasse-III-Syndrom. Untersucht wurden prätherapeutisch ausgefüllte Anamnesebögen, Fotos, Fernröntgenaufnahmen und Modelle. Die Unterschiede zwischen Früh- (Patienten jünger als 9 Jahre) und Spätbehandelten (Patienten älter als 9 Jahre, aber vor dem pubertären Wachstumsspurt)wurden durch Datenerhebung bei Therapieende und einer Abschlussanalyse etwa 25 Jahre nach Therapie analysiert.

**Ergebnisse:**

Die Frühtherapie hatte in 74%, die Spättherapie in 67% der Fälle Erfolg, bei männlichen Patienten gab es mehr Misserfolge. Die posttherapeutischen, skelettalen Werte der Kiefergrößen hatten in der Spätbehandlungsgruppe deutlich größere Werte als in der Frühbehandlungsgruppe. Weiter zeigte sich die skelettale Diskrepanz zwischen Ober-und Unterkiefer in der Spätbehandlungsgruppe stärker ausgeprägt. Die Unterkieferlänge, Cond–Pog, Ramushöhe, APDI, die vordere untere Gesichtshöhe und der Gonion-Winkel waren bei den Spätbehandelten nach der Therapie (T1) größer. Der Winkel AB-Linie/Mandibularebene war bei den Spättherapierten zu allen 3 Zeitpunkten kleiner. Die Camouflagestellung der Unterkieferfrontzähne zu Therapiebeginn (T0) war bei der Spättherapie deutlicher ausgeprägter. Der negative Overjet war in der Frühbehandlungsgruppe anfangs ausgeprägter und zeigte zu Therapieende (T1) eine stärkere Korrektur.

**Schlussfolgerung:**

Die Frühbehandlung der Klasse III bewirkte größere skelettale Veränderungen mit weniger dentaler Kompensation.

## Introduction

Already in early childhood, the growth of the skull reaches a very advanced stage that will only be followed by limited additional growth changes of certain structures in later years [20–22]. Therefore, to optimize skeletal outcome, it appears useful to perform treatment of Angle class III early during the primary or early mixed dentition stage. Several studies [5, 12] have described greater skeletal and dental changes toward Class I by early orthodontic treatment than by later treatment, with early treatment resulting in gonial angle values similar to that found in Class I individuals while, in cases of late treatment, the skeletal disharmony was successfully corrected by camouflage [12]. Some amount of compensation for the differential growth of the maxilla and mandible occurs by the growth taking place at the spheno-occipital synchondrosis of the posterior cranial base. This growth—and the angle between the anterior and posterior cranial base—is capable of influencing the development of Class III [13, 14].

Several authors recommended that chincup treatment of Class III cases should already be performed in the primary or early mixed  dentition stage [5, 8, 16, 19, 25]. Wendell et al. [26] suggested an age of 5–13 years for treatment. According to Mitani and Fukazawa [13] and Mitani and Sakamoto [14], a chincup influences mandibular growth and morphology despite the underlying genetic control; the original pattern will subsequently return, but its extent will depend on the amount of residual growth and on the change already achieved by treatment. We designed this retrospective study of Class III patients to assess dental and skeletal differences between patient being treated early or late and the treatment-related changes of these parameters over time.

## Materials and methods

Pre- and posttreatment anamnestic records, cephalograms, and casts were analyzed for this study, which comprised 38 female and male Class III patients who had received chincup therapy and were followed up after approximately 25 years. Only patients for whom complete pretreatment (T0), posttreatment (T1), and follow-up (T2) documentation was available and who had presented skeletal and dental Class III syndrome at T0 (negative overjet, Wits appraisal <−1 mm, negative ANB difference, Class III malocclusion) were included. Cleft disease or any other syndromes led to exclusion. The patients were required to wear the chincup at 600 g per side for 24 h/day whenever possible and, once a positive overjet was achieved, overnight.

We assigned the patients to early or late treatment group based on their age at T0 (≤9 years or >9 years but before the pubertal growth spurt). Table 1 lists the 36 linear and angular parameters evaluated on each patient’s T0, T1, and T2 cephalograms for analysis and comparison. Traditional radiographs were used for the T0 and T1 tracings, as digital systems had not been available at that time. The tracings were performed independently by two experienced examiners on transparent tracing paper (item 17-222-11; Dentsply, York, PA, USA). For the T2 follow-up examinations, we used a digital 2D imaging system (ProMax 2D S2; Planmeca, Helsinki, Finland) with a magnification factor of 8%. The intraclass correlation coefficient (ICC) for errors of measurement, tracing and assignment committed by the two examiners was 0.986, thus, indicating high agreement.

**Tab. 1:** Cephalometric parameters for the tracings **Tab. 1** Parameter für die Fernröntgendurchzeichnung

Wits	mm
GH	%
SNA	°
SNB	°
ANB	°
Ar-Go-Me	°
Börk’s sum	°
Gn/SN	°
Spp-Spa	mm
Cond-A	mm
Cond-Gn	mm
MM differential	mm
S-N	mm
Go-Me	mm
MaxP/MandP	°
MaxP/SN	°
Go-Me/SN	°
Ar-Go	mm
AB/MandP	°
Cond-Pog/FH	°
APDI	°
Me-Go-N	°
FH/S-Gn	°
Cond-Pog	mm
Cranial base angle	°
AB/facial plane	°
Ant:post cranial b.	Ratio
NS/Gn	°
AB/OccP	°
Spa-Me	mm
Upper gonial angle	°
Upper-incisor incl.	°
Lower-incisor incl.	°
S-N:Spp-Spa	Ratio
Go-Me:Spp-Spa	Ratio
Go-Me:S-N	Ratio

All cephalograms were taken in a standardized fashion, with the help of a cephalostat, and were analyzed in accordance with the principle of Björk, Jarabak, Ricketts, Coben, and McNamara. Additional dental parameters were measured on the casts. Control data of untreated Class III or normal Class I patients were only needed to statistically calculate possible deviations from normal, considering that the study was mainly designed to compare two groups at different times. We therefore relied on normal values from the literature [3, 6, 17], deriving mean values for the relevant age groups. Criteria for treatment success were positive overjet and overbite (≥1 mm) and no transverse crossbite. IBM SPSS Statistics Version 22” (2013) was used for descriptive and explorative statistical analysis of data. Differences were considered significant at *p* ≤ 0.05. We applied a *t* test for independent samples and one-way analysis of variance (ANOVA) to compare mean values and we calculated the ICC for each parameter to determine the tracing precision of the examiners.

## Results

The relationship between the time of treatment and treatment success is shown in Table 2. Outcomes were successful in 74% of cases in the early versus 67% in the late treatment group. Clearly more failures were seen among male patients (80%). However, the early treatment group accounted for two-thirds of all patients. The intergroup differences are shown in greater detail in Table 3. The late treatment group, due to these patient’s more advanced age, showed greater lengths of the maxillary and cranial base already at T0. Also, this group showed higher values for mandibular length, Cond-Pog, ramus height, and lower face height at T0 and T1, larger APDI and gonial angles at T1, smaller angles from AB to mandibular plane at T0, T1, T2, less negative overjet at T0, less positive overjet at T1, and retrusive lower-incisor inclinations at T0 indicating dental compensation.

**Tab. 2:** Interdependence between pretreatment (T0) age and treatment success **Tab. 2** Zusammenhang zwischen dem Behandlungsalter zu Therapiebeginn (T0) und dem Therapieerfolg

All patients			
Age 5–9 years (76%)		Age >9 years (24%)	
Success (74%)	Failure (26%)	Success (67%)	Failure (33%)

**Tab. 3:** Descriptive statistical results (mean values ± SD) divided into early versus late treatment and examination times, including pretreatment (T0), posttreatment (T1), and 25-year follow-up (T2) examinations. Italic numerals of *p* values indicate statistically significant differences between early and late class III treatment at each examination time **Tab. 3** Deskriptive statistische Ergebnisse (Mittelwerte ± SD), eingeteilt nach frühen versus späten Behandlungs- und Untersuchungszeitpunkten - vor Therapie (T0), nach Therapie (T1) und 25-Jahre nach  Therapie (T2). P-Werte in Kursivschrift zeigen statistisch signifikante Unterschiede zwischen früher und später Klasse III Behandlung

		Early treatment group (*n* = 29)						Late treatment group (*n* = 9)						*p* value		
		T0		T1		T2		T0		T1		T2		T0	T1	T2
		Mean	SD	Mean	SD	Mean	SD	Mean	SD	Mean	SD	Mean	SD			
Wits	mm	−3.7	2.5	−1.7	2.4	−2.4	3.3	−3.7	4.1	−3.0	3.2	−4.0	3.3	0.969	0.292	0.318
GH	%	62.4	3.3	64.3	6.7	68.4	5.3	61.3	1.8	65.5	3.1	67.3	2.3	0.452	0.681	0.634
SNA	°	77.9	3.4	78.8	4.0	79.8	4.8	77.2	3.5	77.8	2.8	77.7	2.4	0.634	0.570	0.299
SNB	°	78.1	3.0	78.1	4.1	80.6	4.4	78.3	2.0	79.8	3.7	81.0	4.0	0.865	0.366	0.858
ANB	°	1.9	1.5	2.1	1.6	1.9	2.5	1.5	1.5	2.3	2.0	3.3	2.1	0.578	0.731	0.226
Ar-Go-Me	°	130.4	5.9	125.8	7.5	122.3	6.5	132.3	2.4	127.0	3.7	125.8	1.7	0.452	0.708	*0.044*
Börk’s sum	°	394.5	4.8	388.4	25.2	387.1	18.0	395.2	3.5	376.0	40.6	390.2	3.1	0.747	0.376	0.686
GnSN	°	66.2	4.1	65.7	4.9	65.3	3.9	66.2	1.5	65.8	3.4	64.5	2.9	0.980	0.946	0.661
Spp-Spa	mm	46.7	3.5	50.1	3.6	54.8	4.5	51.2	3.2	53.5	3.1	56.5	3.5	*0.011*	*0.047*	0.415
Cond-A	mm	77.4	4.2	83.8	5.2	90.3	5.2	83.0	4.7	86.3	5.9	89.0	2.6	*0.012*	0.332	0.580
Cond-Gn	mm	107.1	5.8	117.9	8.5	128.8	11.8	116.5	9.1	125.8	13.3	129.5	10.9	*0.007*	0.101	0.897
MM differential	mm	28.9	6.5	33.1	5.2	38.1	10.6	33.5	8.9	39.0	12.1	42.3	10.4	0.188	0.101	0.394
S-N	mm	66.7	3.4	69.9	3.7	74.6	4.5	71.2	3.1	73.7	2.5	75.3	2.5	*0.009*	*0.031*	0.698
Go-Me	mm	61.1	6.1	69.0	6.5	76.9	6.6	69.7	2.9	77.0	5.2	79.0	3.3	*0.003*	*0.012*	0.462
MaxP/MandP	°	27.5	5.0	24.8	5.2	21.8	5.6	27.0	3.5	23.0	3.6	22.3	2.4	0.832	0.431	0.837
MaxP/SN	°	7.6	2.4	7.9	3.3	8.1	2.5	9.0	2.1	7.7	2.6	8.0	2.3	0.202	0.852	0.964
Go-Me/SN	°	34.7	4.0	33.0	6.6	28.6	6.5	36.7	2.9	32.2	4.2	30.0	2.9	0.291	0.776	0.615
Ar-Go	mm	38.1	3.6	42.7	5.2	50.9	5.7	41.0	1.9	49.0	5.3	52.2	5.9	*0.018*	*0.016*	0.639
AB/MandP	°	67.3	4.7	69.9	4.4	68.4	6.6	63.7	3.3	64.7	4.5	63.2	4.5	*0.050*	*0.018*	0.082
Cond-Pog/FH	°	39.3	3.3	42.2	4.0	43.2	3.8	42.7	5.0	44.6	2.2	44.3	2.5	0.071	0.211	0.489
APDI	°	86.0	5.4	84.4	4.5	90.1	5.2	89.8	4.4	92.2	4.5	94.2	5.2	0.132	*0.003*	0.110
Me-Go-N	°	73.5	3.2	72.8	4.9	72.1	5.0	62.7	27.5	73.2	3.5	72.8	1.7	0.090	0.863	0.734
FH/S-Gn	°	50.8	5.1	54.1	4.2	54.9	3.9	55.5	4.1	56.0	3.7	56.0	3.7	*0.043*	0.359	0.546
Cond-Pog	mm	98.0	8.6	108.4	12.2	119.5	15.8	111.0	2.8	122.5	8.2	125.5	5.0	*0.002*	*0.016*	0.373
Cranial base angle	°	120.4	5.1	120.9	3.9	120.9	4.9	122.8	5.5	124.0	4.9	123.3	5.0	0.322	0.121	0.302
AB/facial plane	°	2.6	1.9	2.6	2.1	2.7	2.5	3.3	2.9	3.2	2.3	4.5	1.4	0.500	0.564	0.117
Ant:post cranial b.	Ratio	2.3	0.3	2.2	0.3	2.1	0.2	2.3	0.1	2.1	0.2	2.1	0.3	0.869	0.675	0.867
NS/Gn	°	77.5	6.8	78.4	6.5	80.0	7.7	76.5	6.4	79.3	9.1	78.5	9.2	0.759	0.776	0.694
AB/OccP	°	82.3	4.5	86.2	4.5	83.4	6.8	83.3	6.1	83.5	2.3	83.3	4.5	0.643	0.180	0.991
Spa-Me	mm	57.2	4.8	61.8	6.3	68.3	7.0	60.8	2.7	66.5	3.6	69.8	3.4	*0.034*	*0.037*	0.619
Upper gonial angle	°	56.6	4.6	52.8	4.7	50.2	3.4	57.8	3.5	53.8	1.5	53.0	1.7	0.550	0.603	0.071
Upper-incisor incl.	°	99.8	6.6	106.0	6.0	105.5	10.2	105.3	8.5	109.0	10.0	111.3	9.0	0.108	0.374	0.226
Lower-incisor incl.	°	91.2	7.2	91.7	6.1	93.1	8.2	83.7	2.7	90.3	7.9	94.3	19.1	*0.022*	0.651	0.814
Overbite	mm	0.2	1.6	1.9	1.3	1.6	1.2	−1.0	4.0	1.7	1.0	1.3	2.0	0.289	0.638	0.656
Overjet	mm	−2.3	1.9	2.5	0.8	1.8	0.9	−1.6	4.0	1.7	1.0	0.8	1.9	*0.018*	*0.050*	0.097
Intermolar mand.	mm	39.0	3.1	43.2	1.8	43.0	4.6	40.8	4.9	43.0	3.3	45.0	3.5	0.429	0.899	0.394
Intermolar max.	mm	42.0	2.8	48.1	1.7	48.3	2.5	44.3	3.4	47.9	2.2	49.4	2.7	0.231	0.842	0.416
S-N:Spp-Spa	Ratio	1.4	0.2	1.4	0.1	1.4	0.1	1.4	0.1	1.4	0.1	1.3	0.1	0.883	0.934	0.456
Go-Me:Spp-Spa	Ratio	1.3	0.3	1.4	0.1	1.4	0.1	1.4	0.1	1.4	0.1	1.4	0.1	0.725	0.323	0.923
Go-Me:S-N	Ratio	1.0	0.2	1.0	0.1	1.0	0.1	1.0	0.1	1.0	0.1	1.0	0.0	0.985	0.467	0.494

**Fig. 1:**
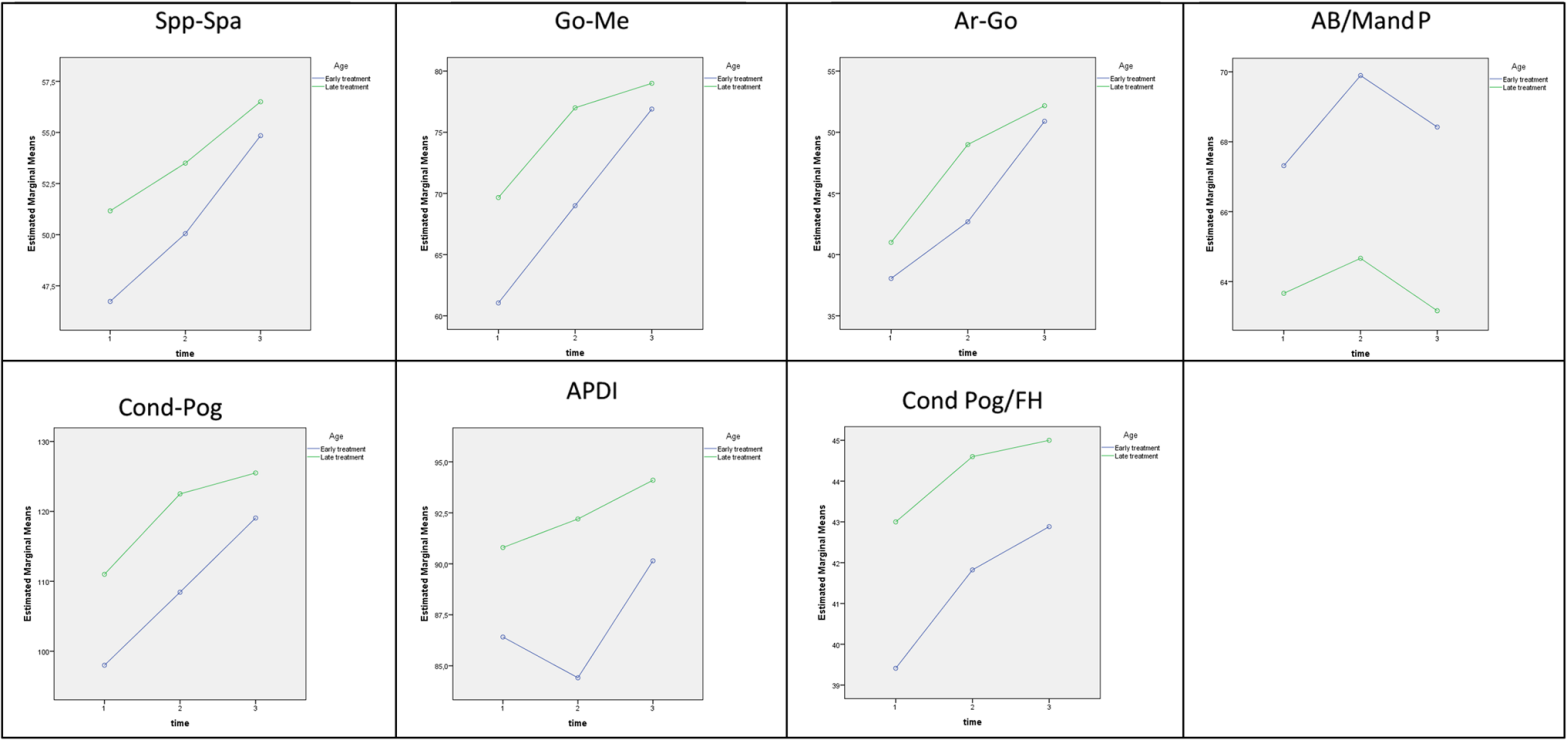
Between-subject effects (age) **Abb. 1** Inter-Subjekt-Effekte (Alter)

**Fig. 2:**
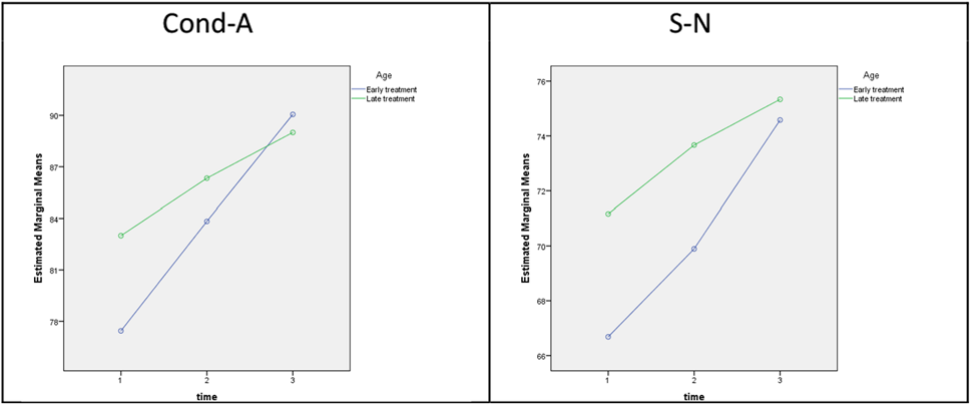
Within-subject effects (age × time) **Abb. 2** Inner-Subjekt-Effekte (Alter × Zeit)

The intergroup differences based on a linear model with repeated measurements, which yields fewer significant differences by looking at the observation period T0, T1, and T2 in its entirety, are summarized in Table 4. Based on the between-subject effect (age), very similar increases over time are seen in the table, but the distances between both ascending curves were significantly different (Fig. 1). Based on the within-subject effect (age × time), significantly different increases in Cond-A und S-N were seen between the two patient groups over time (Fig. 2). Table 5 lists the 95% confidence intervals (CI) and Table 6 the differences in mean values between T0, T1, and T2. The patients in the early treatment group showed more growth overall due to their younger age. These changes included more pronounced mandibular growth from T1 to T2, although with the absolute values being clearly lower than in the late treatment group. Similar growth developments were also noted for the maxilla and cranial base. No significant intergroup difference was, however, seen based on the absolute values at T2.

**Tab. 4:** Significant results (mean values ± SD) broken down by early versus late class III treatment and examination times, including pretreatment (T0), posttreatment (T1), and 25-year follow-up (T2) examinations. The *p* values on the right indicate statistically significant differences between early and late treatment based on a linear model with repeated measurements for between-subject (age) and within-subject (age × time) effects **Tab. 4** Signifikante Ergebnisse (Mittelwerte ± SD)  aufgeschlüsselt  nach Klasse III  Früh- und Spätbehandlungsguppen zu den Zeitpunkten T0, T1, T2. Die p-Werte auf der rechten Seite zeigen statistisch signifikante Unterschiede zwischen der frühen und späten Behandlungsgruppe auf der Grundlage eines linearen Modells mit wiederholten Messungen (Zwischensubjekteffekt (Alter) und Innersubjektefekt  (Alter × Zeit))

		Early treatment group (*n* = 29)						Late treatment group (*n* = 9)						*p* value	
		T0		T1		T2		T0		T1		T2		Age	Age × time
		Mean	SD	Mean	SD	Mean	SD	Mean	SD	Mean	SD	Mean	SD		
Spp-Spa	mm	46.7	3.5	50.1	3.6	54.8	4.5	51.2	3.2	53.5	3.1	56.5	3.5	0.035	
Cond-A	mm	77.4	4.2	83.8	5.2	90.3	5.2	83.0	4.7	86.3	5.9	89.0	2.6		0.008
S-N	mm	66.7	3.4	69.9	3.7	74.6	4.5	71.2	3.1	73.7	2.5	75.3	2.5		0.008
Go-Me	mm	61.1	6.1	69.0	6.5	76.9	6.6	69.7	2.9	77.0	5.2	79.0	3.3	0.008	
Ar-Go	mm	38.1	3.6	42.7	5.2	50.9	5.7	41.0	1.9	49.0	5.3	52.2	5.9	0.056	
AB/MandP	°	67.3	4.7	69.9	4.4	68.4	6.6	63.7	3.3	64.7	4.5	63.2	4.5	0.032	
Cond-Pog/FH	°	39.3	3.3	42.2	4.0	43.2	3.8	42.7	5.0	44.6	2.2	44.3	2.5	0.041	
APDI	°	86.0	5.4	84.4	4.5	90.1	5.2	89.8	4.4	92.2	4.5	94.2	5.2	0.015	
Cond-Pog	mm	98.0	8.6	108.4	12.2	119.5	15.8	111.0	2.8	122.5	8.2	125.5	5.0	0.028

**Tab. 5:** Overview of the 95% confidence intervals associated with the descriptive results, again broken down by early versus late treatment and examination times, including pretreatment (*T0*), posttreatment (*T1*), and 25-year follow-up (*T2*) examinations **Tab. 5** Übersicht über die mit den deskriptiven Ergebnissen verbundenen 95%-Konfidenzintervalle, aufgegliedert nach frühem bzw. spätem Behandlungsbeginn  für die Untersuchungszeitpunkte: vor Therapie (*T0*), nach Therapie (*T1*) und 25-Jahre nach Therapie (T2)

		Early treatment group (*n* = 29)			Late treatment group (*n* = 9)		
		T0	T1	T2	T0	T1	T2
Wits	mm	−4.9 | −2.6	−2.8 | −0.6	−3.9 | −0.9	−7.0 | −0.4	−5.6 | −0.4	−6.7 | −1.3
GH	%	60.9 | 63.9	61.3 | 67.3	66 | 70.8	59.9 | 62.7	63.0 | 68.0	65.5 | 69.2
SNA	°	76.4 | 79.5	77.1 | 80.6	77.7 | 82.0	74.3 | 80.0	75.6 | 80.1	75.7 | 79.6
SNB	°	76.7 | 79.5	76.3 | 79.9	78.6 | 82.6	76.8 | 79.9	76.9 | 82.8	77.8 | 84.2
ANB	°	1.2 | 2.6	1.3 | 2.8	0.8 | 3.1	0.3 | 2.7	0.8 | 3.9	1.7 | 5.0
Ar-Go-Me	°	127.8 | 133.1	122.4 | 129.1	119.4 | 125.2	130.4 | 134.3	124.0 | 130.0	124.5 | 127.2
Börk’s sum	°	392.3 | 396.6	377.0 | 399.7	379.0 | 395.2	392.3 | 398.0	343.5 | 408.5	387.7 | 392.7
GnSN	°	64.4 | 68.0	63.5 | 67.9	63.5 | 67.0	65.0 | 67.3	63.1 | 68.6	62.2 | 66.8
Spp-Spa	mm	45.2 | 48.3	48.4 | 51.7	52.8 | 56.8	48.6 | 53.7	51.0 | 56.0	53.7 | 59.3
Cond-A	mm	75.5 | 79.4	81.4 | 86.2	87.9 | 92.6	79.2 | 86.8	81.6 | 91.0	86.9 | 91.1
Cond-Gn	mm	104.4 | 109.8	114 | 121.8	123.5 | 134.1	109.2 | 123.8	115.2 | 136.5	120.8 | 138.2
MM differential	mm	26.0 | 31.9	30.6 | 35.5	33.3 | 42.8	26.3 | 40.7	29.3 | 48.7	34.0 | 50.6
S-N	mm	65.1 | 68.2	68.2 | 71.6	72.6 | 76.6	68.7 | 73.6	71.7 | 75.7	73.3 | 77.3
Go-Me	mm	58.3 | 63.8	66.1 | 71.9	73.9 | 79.9	67.3 | 72.0	72.9 | 81.1	76.4 | 81.6
MaxP/MandP	°	25.2 | 29.7	22.5 | 27.2	19.3 | 24.3	24.2 | 29.8	20.1 | 25.9	20.4 | 24.3
MaxP/SN	°	6.5 | 8.6	6.5 | 9.4	6.9 | 9.2	7.3 | 10.7	5.6 | 9.7	6.2 | 9.8
Go-Me/SN	°	32.9 | 36.5	30.0 | 36.0	25.6 | 31.5	34.3 | 39	28.8 | 35.5	27.7 | 32.3
Ar-Go	mm	36.4 | 39.7	40.4 | 45.0	48.3 | 53.4	39.5 | 42.5	44.7 | 53.3	47.5 | 56.9
AB/MandP	°	65.2 | 69.4	67.9 | 71.9	65.5 | 71.4	61.0 | 66.3	61.1 | 68.3	59.5 | 66.8
Cond-Pog/FH	°	37.8 | 40.8	40.3 | 44.0	41.4 | 44.9	38.7 | 46.7	42.7 | 46.5	42.3 | 46.3
APDI	°	83.5 | 88.5	82.3 | 86.5	87.7 | 92.5	86.3 | 93.4	88.3 | 96.1	89.9 | 98.2
Me-Go-N	°	72.1 | 75.0	70.6 | 75.0	69.8 | 74.4	71.1 | 76.2	70.4 | 76.0	71.5 | 74.2
FH/S-Gn	°	48.4 | 53.1	52.1 | 56	53.1 | 56.7	52.2 | 58.8	52.7 | 59.3	53.0 | 59.0
Cond-Pog	mm	94.0 | 102.0	102.8 | 114.1	112.4 | 126.6	108.7 | 113.3	115.9 | 129.1	121.5 | 129.5
Cranial base angle	°	118.1 | 122.7	119.2 | 122.6	118.7 | 123.1	118.5 | 127.2	120.1 | 127.9	119.3 | 127.4
AB/facial plane	°	1.8 | 3.6	1.6 | 4.0	1.5 | 4.0	0.3 | 6.4	0.7 | 5.6	3.1 | 6.0
Ant:post cranial b.	Ratio	2.1 | 2.4	2.0 | 2.3	2.0 | 2.2	2.2 | 2.4	1.9 | 2.3	1.9 | 2.3
NS/Gn	°	74.4 | 80.5	75.4 | 81.3	76.5 | 83.5	71.4 | 81.6	72.1 | 86.6	71.1 | 85.9
AB/OccP	°	80.3 | 84.3	84.1 | 88.2	80.3 | 86.4	78.4 | 88.2	81.6 | 85.4	79.8 | 86.9
Spa-Me	mm	55.1 | 59.3	59.0 | 64.6	65.1 | 71.5	58.7 | 63.0	63.6 | 69.4	67.1 | 72.5
Upper gonial angle	°	54.5 | 58.7	50.7 | 54.9	48.7 | 51.8	55 | 60.6	52.7 | 55.0	51.7 | 54.3
Upper-incisor incl.	°	96.8 | 102.8	103.3 | 108.7	100.9 | 110.1	98.5 | 112.2	101 | 117	104.1 | 118.5
Lower-incisor incl.	°	87.9 | 94.4	89.0 | 94.5	89.4 | 96.7	81.5 | 85.8	84 | 96.6	79.1 | 109.6
Overbite	mm	−0.5 | 1.0	1.4 | 2.5	1.1 | 2.2	−4.2 | 2.2	0.8 | 2.5	−0.2 | 2.9
Overjet	mm	−0.8 | 1.0	2.1 | 2.9	1.4 | 2.2	0.0 | 6.3	0.8 | 2.5	−0.7 | 2.4
Intermolar mand	mm	37.4 | 40.7	41.9 | 44.5	40.6 | 45.4	35.3 | 46.3	39.2 | 46.8	41.9 | 48.0
Intermolar max	mm	40.3 | 43.6	46.9 | 49.3	47.0 | 49.6	40.4 | 48.2	45.4 | 50.4	47.1 | 51.8
S-N:Spp-Spa	Ratio	1.3 | 1.5	1.3 | 1.5	1.3 | 1.4	1.3 | 1.5	1.3 | 1.5	1.3 | 1.4
Go-Me:Spp-Spa	Ratio	1.2 | 1.4	1.3 | 1.4	1.3 | 1.5	1.3 | 1.4	1.3 | 1.5	1.3 | 1.5
Go-Me:S-N	Ratio	0.9 | 1.1	0.9 | 1.1	1.0 | 1.1	0.9 | 1.0	1.0 | 1.1	1.0 | 1.1

**Tab. 6:** Developments in the early and late class III treatment groups from T0 to T1 and from T1 to T2. Data are expressed as mean values and standard deviations (SD) and include pretreatment (T0), posttreatment (T1), and 25-year follow-up (T2) data **Tab. 6** Entwicklungen in den frühen und späten Klasse III Therapiegruppen von T0 nach  T1 und von T1 nach T2. Die Daten werden als Mittelwerte und Standardabweichungen (SD) dargestellt.  (vor Therapie =T0, nach Therapie =T1 und 25-Jahre nach  Therapie =T2)

		Early treatment group (*n* = 29)						Late treatment group (*n* = 9)					
		T1–T0		T2–T0		T2–T1		T1–T0		T2–T0		T2–T1	
		Mean	SD	Mean	SD	Mean	SD	Mean	SD	Mean	SD	Mean	SD
Wits	mm	1.9	3.2	1.1	3.6	−0.7	3.5	1.2	6.0	−0.3	6.0	−1.5	0.8
GH	%	1.9	5.4	6.0	4.0	4.1	3.5	4.2	1.9	6.0	1.3	1.8	1.6
SNA	°	0.9	3.0	1.9	3.8	1.0	2.9	0.7	3.6	0.5	4.3	−0.2	2.5
SNB	°	0.0	2.7	2.5	3.2	2.5	2.8	1.5	3.5	2.7	3.8	1.2	2.8
ANB	°	0.2	1.7	0.1	2.9	−0.1	2.6	0.8	1.7	1.8	2.0	1.0	1.7
Ar-Go-Me	°	−4.6	6.7	−8.1	7.4	−3.5	6.2	−5.3	4.6	−6.5	3.4	−1.2	2.6
Börk’s sum	°	−6.1	26.5	−7.4	17.8	−1.3	27.8	−19.2	42.3	−5.0	2.0	14.2	41.7
GnSN	°	−0.5	4.4	−0.9	4.0	−0.4	4.0	−0.3	2.5	−1.7	1.9	−1.3	1.5
Spp-Spa	mm	3.3	3.5	8.1	4.4	4.8	4.4	2.3	2.7	5.3	1.6	3.0	3.0
Cond-A	mm	6.1	4.2	12.6	4.4	6.2	5.2	3.3	2.9	6.0	3.0	2.7	3.6
Cond-Gn	mm	10.9	7.8	21.3	12.0	10.4	12.0	9.3	8.6	13.0	6.7	3.7	3.4
MM differential	mm	4.1	7.7	8.9	10.8	4.8	9.1	5.5	9.1	8.8	4.4	3.3	7.7
S-N	mm	3.2	1.6	7.9	3.0	4.7	3.5	2.5	1.8	4.2	1.0	1.7	1.4
Go-Me	mm	7.9	5.8	15.8	8.8	7.9	6.2	7.3	5.1	9.3	4.2	2.0	2.8
MaxP/MandP	°	−2.6	5.1	−5.6	6.4	−3.0	4.0	−4.0	3.1	−4.7	1.6	−0.7	2.9
MaxP/SN	°	0.4	3.6	0.5	3.5	0.1	2.5	−1.3	2.1	−1.0	2.5	0.3	2.3
Go-Me/SN	°	−1.7	5.9	−6.2	5.9	−4.4	5.8	−4.5	4.1	−6.7	3.4	−2.2	3.9
Ar-Go	mm	4.6	6.1	12.8	5.0	8.2	5.9	8.0	4.6	11.2	4.5	3.2	2.9
AB/MandP	°	2.6	4.2	1.1	5.3	−1.5	3.2	1.0	5.0	−0.5	5.0	−1.5	1.6
Cond-Pog/FH	°	2.8	5.2	3.5	5.6	1.1	3.2	1.6	4.0	1.7	4.1	0.4	0.5
APDI	°	−1.6	5.9	3.7	6.2	5.7	5.7	1.4	2.4	4.3	4.9	1.9	2.7
Me-Go-N	°	−0.7	3.9	−1.4	4.6	−0.7	3.4	−0.5	2.4	−0.8	2.6	−0.3	2.3
FH:SGn	°	3.3	5.6	4.1	6.3	1.4	5.1	1.0	2.5	0.5	3.0	0.6	1.1
Cond-Pog	mm	10.4	9.0	21.1	12.0	10.6	9.8	11.5	7.9	14.5	5.6	3.0	4.9
Cranial base angle	°	0.5	3.2	0.5	4.7	0.0	4.2	1.2	6.1	0.5	6.5	−0.7	1.2
AB/facial plane	°	−0.1	2.4	0.1	2.7	0.2	3.4	−0.2	3.2	1.2	2.5	1.3	1.9
Ant:post cranial b.	Ratio	−0.1	0.2	−0.2	0.2	−0.1	0.2	−0.2	0.2	−0.2	0.2	0.0	0.1
NS/Gn	°	0.9	3.8	2.5	4.0	1.6	3.9	2.8	4.7	2.0	5.3	−0.8	2.0
AB/OccP	°	3.9	4.3	1.1	8.1	−2.8	6.6	0.2	6.2	0.0	8.0	−0.2	4.2
Spa-Me	mm	4.6	3.5	11.1	6.2	6.5	6.3	5.7	4.1	9.0	3.8	3.3	4.7
Upper gonial angle	°	−3.8	5.6	−6.4	5.0	−2.6	3.7	−4.0	4.0	−4.8	4.0	−0.8	1.2
Upper-incisor incl.	°	6.2	8.7	5.7	10.6	−0.5	8.3	3.7	11.6	6.0	10.5	2.3	4.2
Lower-incisor incl.	°	0.6	6.1	1.9	7.7	1.3	6.3	6.7	8.6	10.7	21.1	4.0	16.9
Overbite	mm	1.7	2.4	1.4	1.4	−0.3	1.9	2.7	4.3	2.3	3.8	−0.4	1.2
Overjet	mm	4.8	2.3	4.1	2.4	−0.7	1.1	3.3	4.3	2.4	5.3	−0.9	1.6
Intermolar mand	mm	4.9	2.7	4.2	3.7	0.7	0.8	3.0	2.8	2.9	2.9	1.9	1.3
Intermolar max	mm	5.9	2.1	6.4	2.5	−0.4	3.5	4.9	2.8	4.3	3.5	2.3	2.0
S-N:Spp-Spa	Ratio	0.0	0.2	0.0	0.2	0.0	0.2	0.0	0.0	−0.1	0.1	0.0	0.1
Go-Me:Spp-Spa	Ratio	0.1	0.3	0.1	0.3	0.0	0.1	0.1	0.1	0.0	0.0	0.0	0.1
Go-Me:S-N	Ratio	0.0	0.2	0.0	0.2	0.0	0.1	0.1	0.1	0.1	0.1	0.0	0.0

Table 7 lists only those parameters for which significant differences were obtained between the late versus the early class III treatment groups relative to normal Class I values [6]. The late treatment group, at T1, showed higher values of the skeletal jaw parameters, greater skeletal discrepancies between the maxilla and mandible, higher APDI values by 7.8°, overall, some significantly increased vertical parameters (face-height relationship, gonial angle, upper gonial angle, angle from SN to mandibular plane) and steeper lower-incisor inclination by 9° relative to the Class I normal value at T0. Table 8 compares the 95% CI in both groups to the mean values of untreated Class III patients [3, 17]. The late and the early class III treatment groups showed more regular jaw relationships (ANB) than those untreated patients at T1 and T2. The early treatment group showed clearly lower values for Wits appraisal and (unlike the late treatment group) mandibular length—as well as compensation by the lower incisors—at T1 and T2. The less late and the early class III treatment group showed smaller amounts of lower face height, notably compared to the males among the untreated Class III patients. In both groups, the jaw-base angle was decreased at T2.

**Tab. 7:** Parameters showing significant differences between the early versus late treated Class III group compared to age-matched normal Class I individuals [6]. Results are expressed as *p* values **Tab. 7** Signifikante Unterschiede (dargestellt in p-Werten) bei Vergleich der Klasse III Früh-/Spätbehandelten mit den Normwerten der Klasse I [6]

		T0	T1	T2
GH	%	0.001		
Ar-Go-Me	°	0.008		0.044
Spp-Spa	mm	0.011	0.047	
Cond-A	mm	0.014		
MM differential	mm		0.026	
S-N	mm		0.014	
Go-Me	mm		0.012	
Go-Me/SN	°	0.019		
Ar-Go	mm		0.016	
AB/MandP	°	0.057	0.018	0.047
APDI	°		0.003	
FH/SGn	°	0.043		
Cond-Pog	mm	0.002	0.007	
Spa-Me	mm		0.037	
Upper gonial angle	°			0.015
Lower-incisor incl.	°	0.005

**Tab. 8:** Comparison of the 95% confidence intervals with values reported for age-matched untreated Class III patients of both genders [3, 17]. Results are expressed as mean values for the untreated Class III cases. An upward or downward arrow indicates that  the confidence interval for the late and early treated class III groups is higher or lower than the mean value, respectively **Tab. 8** Vergleich des 95% Konfidenzintervalles mit den  Mittelwerten unbehandelter Klasse III Patienten ( beide Geschlechter) [3, 17]. Die Ergebnisse werden als Mittelwerte für die unbehandelte Klasse III dargestellt. Die Pfeilrichtung beschreibt jeweils ein höheres oder niedrigeres Konfidenzintervall der errechneten Werte für die Klasse III Früh/ Spätbehandlungsgruppen

		Early treatment cases (*n* = 29)						Late treatment cases (*n* = 9)					
		T0		T1		T2		T0		T1		T2	
		Female	Male	Female	Male	Female	Male	Female	Male	Female	Male	Female	Male
Wits	mm	−4.20	−4.40	↓−5.10	↓−4.40	↓−5.70	↓−5.90	−4.75	−4.95	−5.10	−4.40	−5.70	−5.90
SNA	°	↓80.28	↓80.20	↓80.85	↓81.00	80.70	81.10	↓80.21	↓80.10	↓80.85	81.00	↓80.70	↓81.10
SNB	°	↑79.33	↑79.63	↓80.85	↓79.95	81.20	82.40	↓81.04	79.80	80.85	79.95	81.20	82.40
ANB	°	↑0.75	↑0.58	↑0.00	↑0.65	↑−0.50	↑−1.30	↑0.28	0.34	↑0.00	↑0.65	↑−0.50	↑−1.30
Cond-A	mm	↓80.27	↓82.15	↓89.60	↓90.50	90.40	↓94.10	84.04	86.50	89.60	90.50	90.40	94.10
Cond-Gn	mm	104.93	107.88	↓123.35	↓125.70	126.70	↓137.70	111.37	↓131.22	123.35	125.70	126.70	137.70
MM differential	mm	↑24.40	↑25.30	32.30	32.00	36.30	41.00	26.60	28.30	32.30	32.00	36.30	41.00
S-N	mm	67.67	↓70.18	↓71.70	↓74.60	↑72.40	↓77.30	69.47	71.40	71.70	74.60	↑72.40	77.30
MaxP/MandP	°	25.97	26.58	26.35	26.90	↓25.70	↓25.40	25.69	26.99	↓26.35	↓26.90	↓25.70	↓25.40
Cranial base angle	°	122.10	120.78	↓123.00	121.70	123.00	121.80	122.50	121.51	123.00	121.70	123.00	121.80
Spa-Me	mm	↓59.75	↓61.70	↓68.75	↓71.95	71.20	↓77.60	62.01	↓65.57	68.75	↓71.95	71.20	↓77.60
Upper-incisor incl.	°	99.25	99.08	105.45	104.05	105.00	106.10	104.24	102.73	105.45	104.05	105.00	106.10
Lower-incisor incl.	°	88.20	87.30	↑85.80	↑85.90	↑83.90	↑83.60	↓87.80	↓86.00	85.80	85.90	83.90	83.60

## Discussion

Mitani and Fukazawa [13] and Mitani and Sakamoto [14] found that different individuals respond differently to chincup therapy. Uner et al. [24] noticed successful outcomes of chincup treatment, in which overbite and overjet remained unchanged, but with the abnormal growth patterns tending to return to the original position once treatment had been discontinued. Other authors [1], too, were unable to find any statistically significant differences in skeletal and soft-tissue parameters between control and treatment groups except for overjet and overbite at the end of therapy.

Our study revealed distinct treatment effects between the early and late treated Class III groups versus Class I patients and differences between early and late treatment in Class III patients, which we found to persist even approximately 25 years after treatment. Yoshida et al. [28] reported that, compared to Class III patients with a horizontal growth pattern, those with a vertical pattern showed higher pretreatment values for upper and lower face height, total anterior face height, occlusal plane, and gonial angle. After maxillary protraction and chincup treatment, both groups showed increases in SNA, ANB, and upper-jaw size, although with greater ventral displacement in the group with horizontal growth, while no difference existed in mandibular size. We also noted marked upper-jaw growth in both patient groups but, due to the limited number of cases, did not distinguish between growth types at T0.

Sugawara et al. [20] observed in their early treatment group (aged 7 years at T0) a catch-up displacement of the mandible in a forward and downward direction. Ultimately there was no difference between the skeletal profiles in the early and late treatment group. This finding is not confirmed by our study, which demonstrates significant differences between early and late treatment at both T1 and T2. Chincup caused the gonial angle to decrease, improved the SNB and ANB angles, and reduced the lower face height [18]. We also observed these changes, including some significant intergroup differences. The early treatment group showed greater reductions in gonial angle (3.5°) at T2. Reductions in gonial angle were also reported in other studies comparing patients who underwent early or late treatment [4, 9, 10, 11, 19, 25, 26].

Many studies have reported reductions in SNB angle after chincup therapy [5, 23, 24]. We also made this observation but did not find a statistically significant difference in this regard between early and late treatment. SNB increased or decreased by 1° in our late or early treatment group, respectively, and SNA improved by around 1° in the long-term comparison in the early treatment group. The values for mandibular length and ramus height were distinctly higher in the late treatment group. The influence on ramus height, with a difference of approximately 6 mm, seems to be important in this context, since a horizontal growth direction has a negative effect on the prognosis of Class III. The values for lower anterior face height were higher by 4.7 mm in the late compared to the early treatment group. This difference was also found in previous studies [2, 24].

We observed dental compensation mainly of the lower incisors, whose inclination was 83.7° in the late and 91.2° in the early treatment group. This is consistent with previous studies, which also indicated more dental compensation in late treatment groups [2, 24]. The values we measured for APDI, which is a good marker for Class III development, were clearly (by 7.8°) higher in the late treatment group. APDI, maxillomandibular differential, and ramus height are known to be good prognostic parameters for failure [27] and were clearly more pronounced in the late treatment group. Mandibular length, a parameter not readily influenced by treatment, showed higher values in the late treatment group at all three times (T0, T1, and T2).

Especially important about our study is its extremely long follow-up, with T2 following up treatment by approximately 25 years. The data emerging for our sample of Class III patients from this long-term observation can be used as a reference for further studies. However, our data should be interpreted with due consideration given to our limited number of cases, our use of literature-based data for untreated patients, and our retrospective study design [15].

Treatment with a facemask can likewise achieve favorable changes in maxillary and mandibular shape and size—parameters that again were more pronounced in cases of early treatment, which also revealed favorable growth changes in both jaws whereas late treatment influenced mandibular growth only [7, 8]. While Yüksel et al. [29] reported contrary observations of no significant differences between early and late treatment with a facemask, they did achieve improvements in overjet, SNB angle, maxillomandibular differential, Con-A, and Wits appraisal compared to a control group.

## Conclusions

Early initiation is an important prerequisite for successful outcome in the treatment of Class III syndrome. Compared to the outcome of late treatment, those of early treatment are characterized by significant skeletal changes, most importantly in terms of mandibular length, ramus height, and growth direction (gonial angle). Early treatment results in a better jaw relationship and less dental compensation.
